# Specific Avenin Cross-Reactivity with G12 Antibody in a Wide Range of Current Oat Cultivars

**DOI:** 10.3390/foods11040567

**Published:** 2022-02-16

**Authors:** Václav Dvořáček, Anna Kotrbová-Kozak, Jana Kozová-Doležalová, Michal Jágr, Petra Hlásná Čepková, Pavel Vítámvás, Klára Kosová

**Affiliations:** Crop Research Institute, Drnovská 507/73, 16106 Prague, Czech Republic; dvoracek@vurv.cz (V.D.); anna.kotrbova@vurv.cz (A.K.-K.); dolzealovaj@vurv.cz (J.K.-D.); jagr@vurv.cz (M.J.); hlasna@vurv.cz (P.H.Č.); vitamvas@vurv.cz (P.V.)

**Keywords:** oat, cultivars, avenins, gluten epitopes, celiac disease, ELISA, immunoblot

## Abstract

Current clinical studies confirm that the consumption of oats for people suffering from celiac disease is safe. Some studies have confirmed different levels of immunoreactive gluten epitopes of oats in different cultivars, while others explain these differences due to contamination with gluten-rich species or as random cross-reactivity ELISA of homologous oat epitopes with anti-wheat gliadin antibodies. The aim of our two-year study was therefore to map cross-reactive oat epitopes in a set of 132 oat cultivars using a G12-based ELISA kit. The results were focused on the varietal and annual level of cross-reactivity (interference) of avenin epitopes with the G12 antibody on the identification of potential cultivars with significantly different interferences and assessing the degree of risk of possible false-contamination with external gluten. Although repeated evaluations confirmed high year-to-year variability (RSD ≥ 30%) in approximately 2/3 of the cultivars, the content of interfering avenin epitopes with G12 did not exceed the considered safe limit (20 mg·kg^−1^) for celiacs. At the same time, not only annual but, above all, significant cultivar dependences in the interference of avenins to the G12 antibody were demonstrated. Genetic dependence was further confirmed in connection with the proven avenin polymorphism as well as immunoblotting with the identification of interfering peptides with the G12 antibody in the 25 and 30 kDa regions. It was the occurrence of two bands around 30 kDa that predominantly occurred in oat cultivars with a relatively higher content of cross-reactive avenins (12–16 mg·kg^−1^). Due to the fact that the contents of interfering avenins ranged in several cultivars even over 16 mg·kg^−1^, the choice of a suitable oat cultivar may be crucial for gluten-free food producers, as it reduces the risk of a possible false-response of the commercial ELISA kits when checking the real-gluten contamination.

## 1. Introduction

The genus *Avena* includes about 70 species, many of which are commercially cultivated. Most oats produced worldwide belong to the hexaploid species, in which oats (*Avena sativa* L.) are the most economically important hexaploid species (2n = 6x = 42. AACCDD). Part of this species is also the so-called oats with naked grain, taxonomically classified as *Avena sativa* ssp. *nudisativa* L. In the hulled oats (*Avena sativa* L.), we further distinguish two other different forms of cultivars with white resp. black grain [1].

Although oat is still predominantly used as forage and livestock feed, the popularity of oat consumption in the human diet recognizes the increasing trend [2]. Oat grain is an important source of proteins, fat, vitamins, minerals (Fe and Ca), fibers, as well as important bioactive compounds such as β glucans and avenanthramides [3]. Therefore, oat consumption is recommended for all age spectra of the human population [4,5]. Oats are also therapeutically effective against diabetes, high blood pressure, inflammatory conditions, and other diseases [6]. Its nutritional properties and anti-inflammatory effect should therefore also be beneficial for patients suffering from celiac disease (CD) [7].

Until recently, however, there was an ambiguous view in this regard, which in part still persists. CD is an autoimmune disease associated with permanent intolerance to gluten with a prevalence of about 1% in the population [8]. Currently, the only effective treatment is the adherence to a gluten-free diet (GFD) but the recommended amounts of fiber, iron, and calcium can be more difficult to obtain. Thus, supplementing a GFD with oats could potentially diminish nutrient deficiency and may improve the quality of life. The above-mentioned ambiguity is mainly connected with inconsistent outcomes observed in oat clinical studies, some of which have mentioned that some CD patients possess sensitivity to oat proteins [4]. Fritz and Chen [4] stated, based on a meta-analysis of 12 clinical studies, that the reasons for the immunotoxicity of oats for patients with celiac disease may have included cross-contamination of oats with wheat, barley, or rye gluten. The next recent clinical meta-analysis by [5] did not bring any evidence that the addition of oats to a GFD adversely affects symptoms, histology, immunity, or serologic features of patients with celiac disease. Nevertheless, the authors add that another detailed clinical study is needed.

A different cultivar toxicity of oat for celiac patients is mentioned as the next theory explaining the observed variability in clinical symptoms. Comino Montilla et al. [9] and the continuing research of Real et al. [8] confirmed different levels of immunotoxicity in individual oat cultivars based on their genetically different amino acid compositions of isolated avenins. Additionally, Ballabio et al. [10] evaluated the cross-reactivity between avenins and gliadins by both SDS-PAGE/immunoblotting and ELISA methods in 36 oat cultivars. In most oat samples, the content of cross-reactive proteins measured by ELISA was below 20 ppm, but in a few varieties, it was above 80 ppm.

On the other hand, Gilissen et al. [7] disagreed with the interpretation of the immunogenic responses of the G12 monoclonal antibodies (G12 mAb) in the case of oat and detected immunochemical differences designated as misleading results. According to these authors, this is due to cross-reactivity with some homologous sequences in avenins, which may not correspond to clinically validated toxic sequences. This is already indicated by the results of Londono et al. [11], which confirmed that no perfect recognition sites for the applied R5 or G12 antibodies are present in oat avenins. Thus, these antibodies promiscuously recognize some variants of the oat avenin protein sequence that appear to have one or two amino acid substitutions at the recognized sites. Due to their false reactivity, these commercial ELISA tests cannot objectively differentiate between more resp. and less reactive oat cultivars.

According to Lexhaller et al. [12], however, there are also problems with the quantification of standard gluten using commercial ELISA kits. The authors found that different ELISA kits provide variable results depending on the source of gluten (wheat, rye, or barley) and the gluten fraction (prolamins or glutelins). They revealed that gluten content was up to six-folds overestimated in the case of rye and up to seven-folds underestimated in the case of barley. Similarly, Huang et al. [13] reported an overestimation of hordein contents in barley samples with respect to ϖ-gliadin when C hordein was used as a standard for R5 ELISA. It is evident that different gluten sources showed highly variable reactivity with specific kit antibodies. Tanner et al. [14] reported that for the accurate determination of the hordein content in the sample using an ELISA kit, a suitable standard with hordein proteins’ composition identical to the test samples should be employed. It can be concluded that appropriate attention to the preparation of ELISA validation standards for the determination of gluten content has to be paid [15].

Proteomic studies of homologous groups of oat prolamins (so-called avenins) have so far revealed only four immunoreactive epitopes including HLA-DQ2.5-ave-1a “PYPEQQEPF”, HLA-DQ2.5-ave-1b “PYPEQQQPF”, HLA-DQ2.5-ave-1c “PYPEQEQPI”, and HLA-DQ2.5-ave-2 “PYPEQQPF” (Sollid et al. [16]). A phylogenetic analysis of avenin sequences in 13 *Avena* species, including twelve diploid and tetraploid *Avena* species, as well as a hexaploid *Avena* cultivar Gigant, carried out by Londono et al. [11], revealed that oat genotypes usually encode seven to ten avenin genes belonging to four avenin phylogenetic groups. Regarding avenin immunoreactivity, avenin group II contained the epitope DQ2.5-ave-1b “PYPEQQQPF” and avenin group III contained the epitope DQ2.5-ave-1a “PYPEQQEPF”, while avenin genes from groups I and IV contained no T-cell immunoreactive epitopes. However, the authors reported that none of the oat species studied lacked group II or III avenins, i.e., they all possess at least one of the two avenin-specific T cell epitopes. However, it should be kept in mind that the genes present in a given genome can be expressed at differential rates, resulting in different final protein levels; thus, the resulting levels of immunogenic proteins cannot be estimated from the presence of genes or gene copies in the genome due to various mechanisms regulating transcription and post-transcription processes. For more details on genotypic differences in the content of immunoreactive avenins, see the review by Kosová et al. [17]. Therefore, genotypic differences in the accumulation of avenin proteins with gluten-like epitopes were observed by Mujico et al. [18] who found differences in the immunogenic profile within a set of oat varieties and thus concluded that breeding for oat varieties with a low profile of immunogenic proteins may be a realistic aim.

According to the above studies, it can be concluded that the concentration of immunoreactive epitopes in oats seems to be very low, allowing for the consumption of oats for celiacs. Nevertheless, cultivar-specific profiles of avenins containing enhanced levels of cross-reactive proteins, including the above-mentioned immunoreactive epitopes, cannot be completely ruled out. At the same time, the cross-reactivity of antibodies from existing commercially available diagnostic ELISA kits (e.g., G12 and R5) to homologous avenins can cause problems in accurately detecting true-gluten contamination in oat samples. Since R5 antibody was reported to not interact with oat gluten-like proteins by Ahola et al. [19], we have chosen G12 antibody for avenins containing gluten-like sequences based on the reported cross-reactivity.

The aim of the present study was to define the level of the cultivar and annual cross-reactivity (interference) of avenin epitopes with the G12 antibody to identify potential cultivars with significantly lower/higher interference and to assess, in these cases, the level of risk of possible false-gluten contamination.

## 2. Materials and Methods

### 2.1. Plant Material and Field Trials

Analyses were carried out in a wide set of 132 commercial oat cultivars originated from 21 countries including a selected 5 new Czech breeding lines. According to the taxonomical classification, the set included 100 cultivars of *Avena sativa* L., 11 cultivars of *Avena sativa var. nigra* L., and 21 samples of *Avena nuda* L. (Table 1 and Supplementary Table S1). Materials were cultivated in small breeding plots (4.5 m^2^) at the breeding station Selgen a.s., Krukanice, in the Czech Republic over the course of 2 years (2018–2019). Basic weather data related to average daily temperatures and precipitation during a decade period throughout both growing seasons are provided in Supplementary Figure S1. Both seasons were characterized by relatively high temperatures and low precipitation, which led to relatively high grain protein content. Oat grains (min. 200 g) were dehulled (except naked cultivars) and milled with a laboratory grinder (IKA-Werke, Staufen, Germany). Grinding was carried out using an IKA laboratory grinder without an integrated sieve and the particle size did not exceed 0.6 mm. The obtained meal was used for subsequent chemical analyses. Prior to follow-up chemical analyses, selected grain samples were carefully visually inspected to eliminate possible external sources of gluten contamination.

### 2.2. Chemical Analyses

All chemicals and solvents used were of analytical grade. Chemical analyses included immunoassay tests of the gluten content in oat grain and, on the basis of purified avenins, prediction of the crude protein content in oat grain using the NIR spectroscopic method and electrophoretic analysis of avenins.

#### 2.2.1. Determination of Crude Protein Content

Crude protein content (CP) in oat grain was detected using a Nicolet Antaris II Fourier transform spectrophotometer equipped with an interferometer (Thermo Fisher Scientific, Madison, Wisconsin, USA). Approximately 25 g of intact wheat grains was placed on the rotary sample-cup spinner and 64 interferometer sub-scans in the range from 10,000 to 4000 cm^−1^ (wavelength: 1000–2500 nm) and with a resolution of 2 cm^−1^ (0.5 nm) were applied for the collection of each spectrum sample by means of the software Omnic 7.3 (Thermo Fisher Scientific). Parameters of the CP prediction model included: range of calibration for CP (14–21%): correlation coefficient of calibration (r = 0.98); standard error of calibration (SEC = 0.25%); standard error of cross-validation (SECV = 0.58%); and standard error of prediction (SEP = 0.54%). Processing of the collected spectra as well as CP prediction was carried out using TQ analyst^®^ software (company, state).

#### 2.2.2. Immunoassay Tests

Standard measurements in oatmeal were performed by using an ELISA test kit AgraQuant Gluten G12 assay (Romer labs Diagnostics, Tulln an der Donau, Austria) according to the manufacturer’s protocol. All ELISA measurements were in a separate room to avoid gluten contaminations. The absorbance was recorded at 450 nm by using the Sunrise (Tecan) microplate reader [20]. The detection limit (LOD) of the kit according to the manufacturer is 2 mg/kg and the quantification limit (LOQ) is 4 mg/kg. Two independent extractions and four replicates were used per each sample.

A similar immunochemical procedure was applied in the case of purified avenin in the selected 12 oat cultivars with significantly lower concentrations of cross-reactive avenin epitopes (gluten_(G12)_). The resulting concentration of gluten-like homologous peptides per avenin unit (ng mg^−1^) was determined as a ratio between the concentration of reactive gluten epitopes determined by the G12-based kit (ng·mL^−1^) and the concentration of purified avenin (mg·mL^−1^) determined by RP-HPLC (see Section 2.2.5).

#### 2.2.3. Isolation of Pure Avenins

Chilly precipitation of avenins was carried out according to Tanner et al. [21] with some modifications. In total, 0.5 g of oat flour was extracted in 4.5 mL of 50% (*v*/*v*) ethanol, with vortexing regularly over 1 h and centrifuging at 3000× *g* for 1 h. The 50% ethanol supernatants were chilled at 4 °C overnight, centrifuged as above, and the pellets were redissolved in 0.3 mL 60% (*v*/*v*) ethanol during 2 h of incubation at room temperature. The final sample preparation for the above-mentioned immunoassay test included separation of 75 µL of redissolved purified avenins with the addition of the 25 µL of standard extraction buffer of the G12 ELISA kit. The pure avenin thus separated were 50 times the concentrated in volume compared to the standard protein isolation in the manufacturer´s protocol (see Section 2.2.2).

#### 2.2.4. Avenin Profiling by SDS-PAGE

The polymorphism of avenin peptides was studied in the condition of the sodium dodecyl sulfate–polyacrylamide gel electrophoresis (SDS-PAGE) according to Laemmli [22] on 14% separating gels and 4.6% stacking gels in Thermo Scientific™ Owl™ Dual-Gel Vertical Electrophoresis Systems (US) units. Extractable proteins were diluted in the ratio 1:1 (*v*/*v*) with the sample buffer (0.055 M Tris–HCl, pH 6.8; 2% SDS; 40% glycerol; 1% DTT; and 0.0025% bromophenol blue) and heated at 90 °C for 5 min. After the run, the proteins were fixed for 30 min in 12% trichloroacetic acid and stained for 30 min with Coomassie Brilliant Blue R-250. Molecular weights of the polypeptides were estimated by using the Thermo Scientific™ PageRuler™ Unstained Broad Range Protein Ladder.

#### 2.2.5. Pure Avenin Quantification by RP-HPLC

The applied RP-HPLC method was carried out according to Gojković-Cvjetković et al. [23] with the following modifications. The contents and characterization of the isolated avenins were assessed in samples (10 µL) through reverse-phase high-performance liquid chromatography (RP-HPLC) using a Waters 2965 apparatus with both a UV detector (210 nm) and 300 SB-C8 Zorbax Poroshell ™ column (75 mm × 2.1 mm, 5 µm particles) linked to a Zorba 300SB-C8 cartridge guard column (Rockland Technologies, Inc., Newport, DE, USA). The external calibration curve was constructed with PWG gliadin (0.5 to 5 mg·mL^−1^ in ethanol/water). To elute the avenins from the column, 0.1% trifluoroacetic acid (TFA) in deionized water (eluent B) and 0.1% trifluoroacetic acid (TFA) in acetonitrile (ACN) of HPLC purity (eluent A) were applied and mixed to produce the ACN gradient as follows: 0–1 min, 23% A; 1–2.5 min, 23–30% A; 2.5–9.5 min, 30–47% A; 9.5–11 min, 47% A; 11–13 min, 47–23% A; and 13–14 min, 23% A at 1 cm^3^/ min flow. Control of the chromatography and data quantitation was provided by Empower Bulid 1154 software.

#### 2.2.6. Immunoblotting

Avenins that cross-reacted with G12 antibody (Anti-gliadin 33mer G12 Antibody, iVYDAL In Vitro Diagnostics) were isolated by chilly precipitation (see Section 2.2.3) and 4 µg of the extracted proteins from each sample was separated by SDS-PAGE (12.5%; see Section 2.2.4.). The proteins were electrophoretically transferred to nitrocellulose (0.45 mm; Pharmacia Biotech, Uppsala, Sweden). The G12 antibody bound to protein bands were visualized by Goat Anti-Mouse secondary antibody with AP conjugate (Bio-Rad) and BCIP/NBT staining by the manufacturer´s protocol (Bio-Rad, Hercules, CA, USA). The GS-800-calibrated densitometer (Bio-Rad) was used for the image capture of the visualized cross-reacted avenin bands. Quantification of the cross-reacted avenin bands was done using Quantity One version 4.6.2 software (Bio-Rad).

### 2.3. Data Analyses

All statistical analyses (histograms, Pearson’s correlation, graphs constructed on basis of ANOVA, Duncan’s multiple range test, and Tukey’s test of significance) were carried out using statistical software Statistica 7.1 CZ. The percentage comparison of factor effects (e.g., cultivar–C, year–Y, and C–Y interaction) were calculated as their percentage proportion of the Total Sum Square (TSS). The inter-year content variability of homologous gluten peptides (G12) for the individual oat cultivar was calculated as a relative standard deviation (RSD) according to the formula 100·s/$\bar{x}$, where ‘s’ is the inter-year standard deviation of the oat cultivar and $\bar{x}$ is a mean of the gluten_(G12)_ content from years 2018 and 2019.

## 3. Results and Discussion

### 3.1. Contents and Variability of Cross-Reactive Gluten Peptides in the Set of Oat Cultivars

Two-year mean values of cross-reactive gluten (avenin) peptides (Gluten_G12_) and the total content of grain proteins in the set of 132 oat genotypes are given in Table 2. The mean content of the cross-reactive gluten peptides in the whole sample set was 7.2 mg·kg^−1^. None of the tested cultivars’ harvest, except for the French cultivar Sirene from 2018, exceeded the 20 mg·kg^−1^ limit for gluten-free foods safe for celiacs [24]. However, in the 2019 harvest, the cross-reactive avenin content in Sirene was only 9.5 mg·kg^−1^. Due to the achieved results of all the other cultivars in 2018, it must have been an accidental contamination of the Sirene sample. There can be only a few explanations for the repeatedly verified high values of this sample. First of all, contamination by other cereal species was not detected during visual inspection. Furthermore, the Sirene sample contamination during processing, for example, through grinding equipment, storage bags, or contaminated laboratory tools, represents the most probable explanation. A significant difference is evident between the high RSD value found for reactive epitopes (RSD = 46.1%) and the much lower variability in the total proteins (RSD = 6.8%). Almost no relationship between the two parameters is further confirmed by the low negative correlation (r = −0.13). This is in conflict with the relationships known in the so-called Osborne fractionation of proteins; when in cereals, a close relationship is declared between the content of the crude protein and the storage prolamine fraction [25]. The non-specific reaction of avenins with homologous gluten epitopes has already been described by Londono et al. [11]. For example, the avenin-derived sequence QPQLQ revealed significant cross-reactivity to the wheat QPQLPY epitope while the avenin-derived epitopes QPQQQA, QQQQPF, QPQQQA, QQQQPF, QPQQLP, and QPQLPF revealed cross-reactivity to the wheat gluten epitope QPQQPY. Out of the primary antibodies raised against wheat gluten peptides, we have chosen G12 antibody due to the previously reported cross-reactivity with avenins [19]. However, unlike Ahola et al. [19], we were able to detect G12-cross-reactive avenins not only by the ELISA kit but also by immunoblotting.

Statistical analysis of the quantitative content of gluten-like cross-reactive proteins is provided in Table 2.

**Figure 1:**
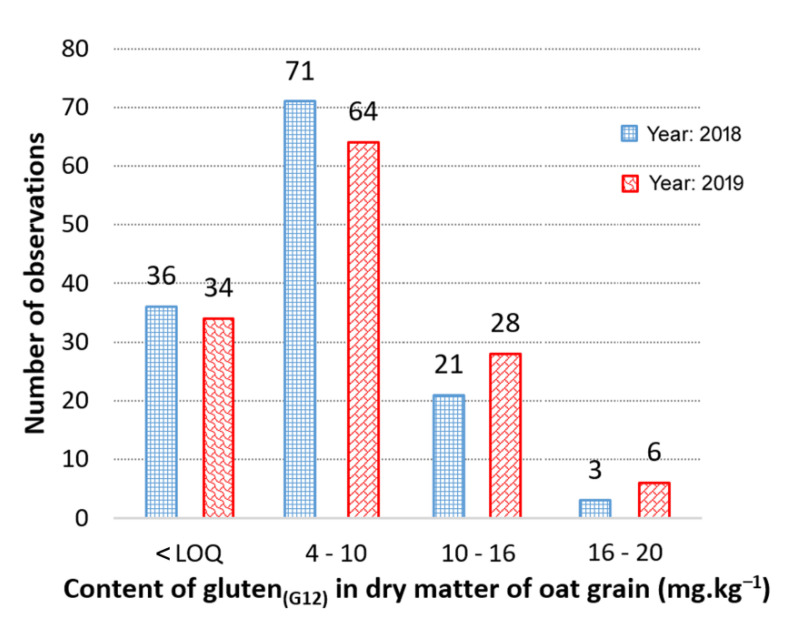
Year distribution of detected average content of cross-reactive avenin peptides (Gluten_G12_) in the set of 132 oat cultivars. Note: The detected mean value 309.4 ppm of Sirene cultivar from 2018 was not included.

The following distribution analysis in the content of cross-reactive gluten peptides confirmed similar annual distributions (Figure 1). The highest frequency of cross-reactive gluten content was found in the range of 4–10 mg·kg^−1^. Towards higher and lower concentrations, the number of cases decreased significantly (Figure 1). However, it has to be noted that the values of the mean, standard deviation, and RSD in the 0–5 mg·kg^−1^ range are significantly affected by the limit of quantification (LOQ = 4 mg·kg^−1^) and by the limit of detection (LOD = 2 mg·kg^−1^) of the kit used for gluten detection (AACC Method 38–52.01). The obtained values of gluten content lower than 4 mg·kg^−1^ could show a higher rate of analytical uncertainty. The values of cross-reactive gluten content lower than LOD (five cultivars in 2018 sampling and nine cultivars in 2019 sampling) were automatically assigned to the 2 mg·kg^−1^ detection limit. This certain inter-year regularity indicated that even clearly uncontaminated gluten-free oats can show a certain fluctuation in the content of detected cross-reactive avenins. In some rare cases (from our data, the relative frequency was 2–5%), their content can even approach the safe limit for celiacs. Consistent with Tanner et al. [21], it can be assumed that these interacting avenin epitopes do not correspond to the epitopes stimulating the immune response in patients with CD. However, in the standard control of gluten-free oat products, it is not possible to distinguish whether it is only an interacting avenin epitope or a possible external contamination with immune-reactive gluten. The observed annual similarity also theoretically suggests a possible genotype specificity of the reactivity of avenin epitopes with the G12 antibody.

**Figure 2:**
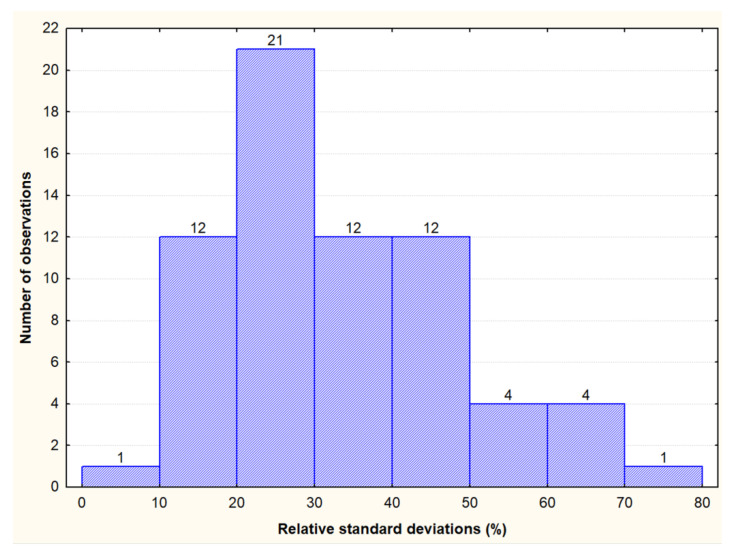
Inter-year distribution of the relative standard deviation (RSD) of the immunoreactive gluten peptides content in a set of 67 oat cultivars with values above 4 mg·kg^−1^ in both years (2018 and 2019). Note: The oat cultivar Sirene was not included.

The total distribution of individual inter-year variability (RSD) in the content of cross-reactive avenins was further calculated only for a set of 67 cultivars, which, in both years (2018 and 2019), showed values above the LOQ (4 mg·kg^−1^). RSD parameters confirmed a wide range of values up to 80% (Figure 2). Inter-year variability of up to 30% was found in only about half of the tested cultivars.

Another hitherto unmentioned factor influencing the content variability of epitopes is also the influence of weather conditions (e.g., temperature stress), which affect the biosynthesis of various fractions of prolamins and thus the abundance of avenins containing immunoreactive gluten-like sequences [25]. The presumed cultivar-specific cross-reactivity is thus just one of the other factors mentioned above that affect the quantification of the gluten content in oats by current commercial kits (e.g., year and cropping system, kit detection limit, random interaction with non-specific avenin epitopes, inability to distinguish external gluten contamination from reaction with avenins, etc.).

### 3.2. Contrasting Groups of Oat Cultivars in the Content of Cross-Reactive Avenin Peptides

In the subsequent analysis, we further focused on the detection of contrasting groups of oat genotypes containing minimum (≤5 mg·kg^−1^) and maximum (≥11 mg·kg^−1^) contents of cross-reactive avenin peptides. At the same time, inter-year variability (RSD < 31%) was also taken into account for the selection of cultivars. The reason was the assumption of possible partial elimination of some “error” factors, including the LOQ kit, and greater random interference with homologous avenine epitopes.

**Figure 3:**
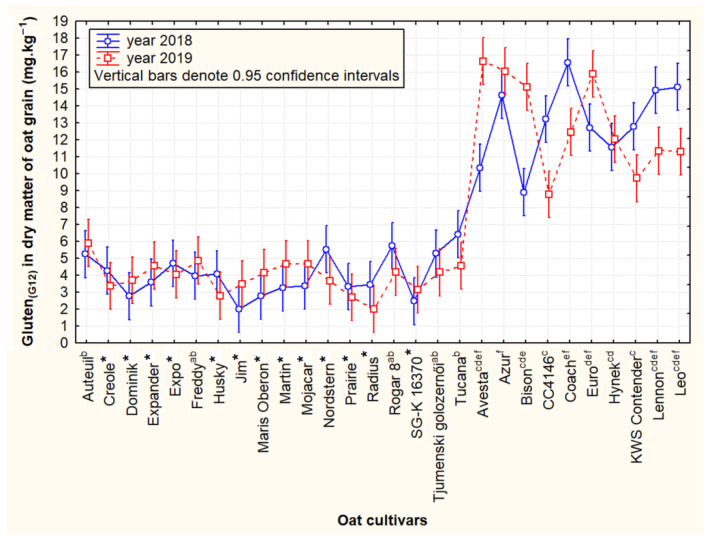
A two-year statistical comparison of selected oat cultivars that showed lower inter-year variability (RSD < 31%) and contrasting average contents of cross-reactive avenin homologous peptides-Gluten_(G12)_ (≤5 vs. ≥11 mg·kg^−1^). Note: Cultivars marked with different letter apostrophes are statistically significant at *p* ≤ 0.05-HSD Tukey’s test. *: Oat cultivars containing cross-reactive avenins in the range of ≥LOD and ≤LOQ of the analytical kit were used to display the graph. Due to the lower accuracy of the analysis, no statistical significance of the cultivars was indicated in these cases.

Statistical analysis indicated a significant difference in the content of reactive avenine peptides detected by monoclonal antibody G12 between the two groups. An accurate interpretation of statistically significant differences between the two groups was not indicated for cultivars under the LOQ kit due to a possible higher determination error (Figure 3). On the other hand, the achieved contents of the cross-reactive avenins in these cultivars can be considered definitely lower than the LOQ due to repeated measurements and thus clearly belong to this group of 18 cultivars with low reactivity.

In the group with the cross-reactive avenin content higher than 11 mg·kg^−1^ (10 cultivars), significant genotypic and inter-year differences were determined as well; it was clearly indicated by non-overlapping confidence intervals among several oat cultivars in both tested years. Detailed data on the grain content of gluten-like proteins in the whole set of oat cultivars tested including both growing seasons are presented in Supplementary Table S2.

The results of our analysis confirmed that despite the relatively high variability in the content of cross-reactive avenins, smaller groups of significantly different cultivars can be found. At the same time, the contents of cross-reactive avenins at around 16 mg·kg^−1^, which are close to the 20 mg kg^−1^ limit for celiacs, were detected in only four cultivar and only in one year (Avesta—2019, Azur—2019, Coach—2018, and Euro—2019). With respect to the fact that the “immunoreactive” avenins thus detected show only sequence homology and cross-reactivity with wheat gluten peptides, it can be concluded that even oat cultivars with a detected level of around 20 mg kg^−1^ will be safe for patients with CD. However, the problem still remains concerning the case of distinguishing external contamination with gluten from the naturally reactive background of oats, which may be even more pronounced in special oat products based on protein concentrates. Producers can thus be advised not to include the avenin (prolamin) fraction in these products not only because of the generally lower nutritional quality of these proteins in cereals, as mentioned by Shewry and Halford [26], but also in the context of the risk of a higher cross-reactive avenin background.

### 3.3. Avenin Protein Polymorphism and Immunoblot Analysis in Groups of Oat Cultivars with Contrasting Cross-Reactivity

**Figure 4:**
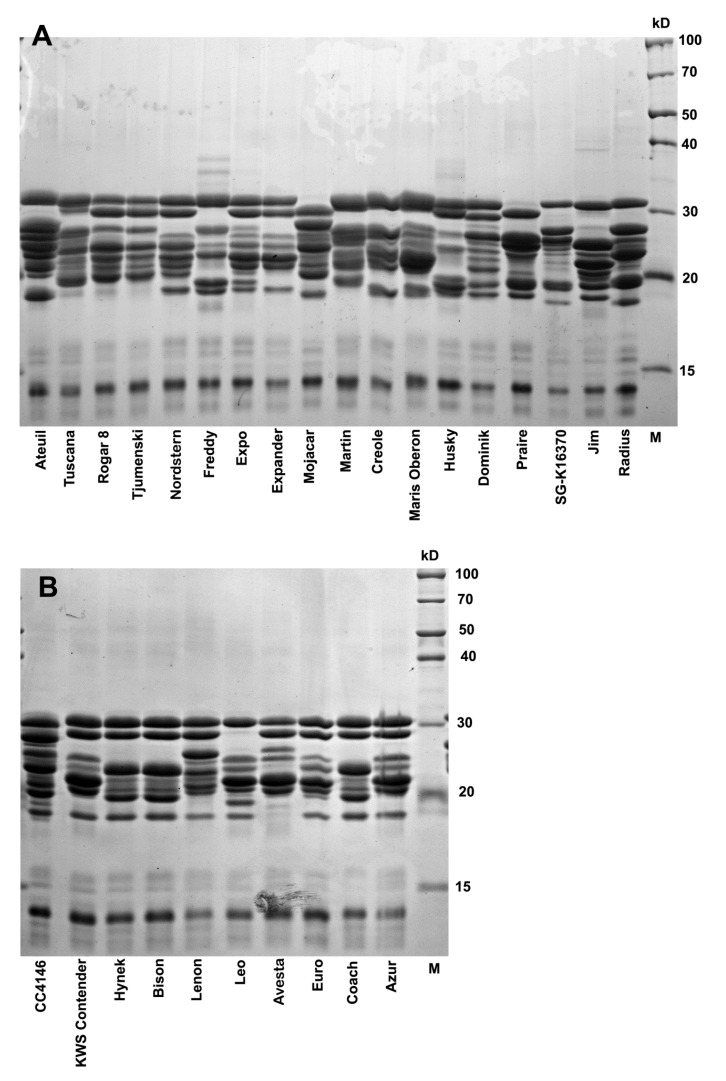
Electrophoretic 14% SDS-PAGE gel visualization of isolated avenin extracts in two groups of oat cultivars with a lower (**A**) and higher gluten_(G12)_ peptide content (**B**).

The peptide polymorphism of the avenin protein fraction (SDS-PAGE) in the cultivars with statistically significantly different reactivity (Figure 3) is shown in Figure 4A,B. Consistent with the results of Tanner et al. [16], five to seven significant bands (peptides) in the range of 20–30 kDa were detected on electrophoretic gels. In accordance with Gregová et al. [27] and Ahola et al. [19], avenin polymorphism is a useful tool for identifying cultivars, but it will also have its limits. This is indicated by identical avenin spectra in some cultivars (e.g., the cultivars Hynek and Bison; see Figure 4B).

**Figure 5:**
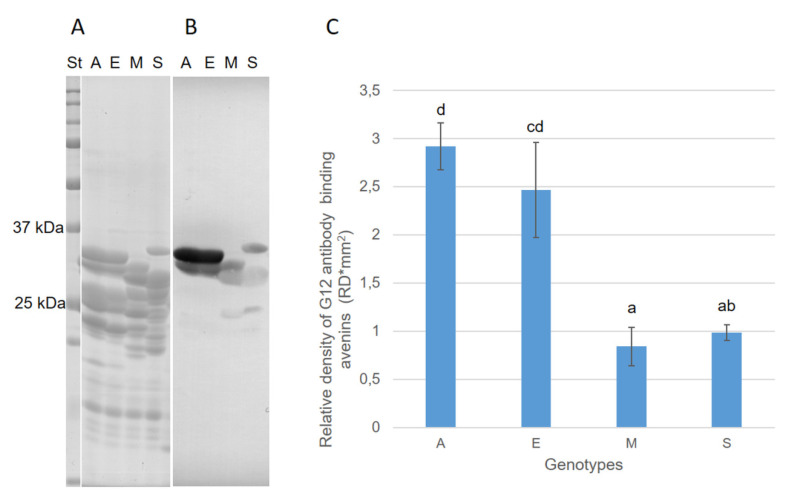
Immunoblotting: (**A**) Coomassie-stained peptides in 12.5% SDS-PAGE gel; (**B**) detection of G12 antibody-reactive bands in nitrocellulose membrane; and (**C**) densitometric analysis of protein reactivity to G12 antibody. Error bars indicate SD and different letters indicate significant differences at 0.05 level using ANOVA analysis followed by Duncan’s multiple range test. A—Azur, E—Euro, M—Mojacar, S—SG-K 16370, and St—All Blue Precision Plus Protein Standard (Bio-Rad).

Immunoblot analysis using G12 antibody on avenin protein profiles extracted from four oat cultivars—Azur, Euro, SG-K 16370, and Mojacar—revealed G12 cross-reactivity with specific motifs in avenins (Figure 5). Significant overloading of pure avenins on SDS electrophoretic gel was intentional here in order to detect the maximum amount of reactive avenin peptides, including possible external gluten contamination. G12 interacted with two avenin bands at around 30 kDa and with some bands at around 25 kDa. In our opinion, possible external contamination with the immunoreactive prolamins of other cereal species can be ruled out since Triticeae gluten prolamins have higher molecular weights (see, e.g., reviews by Wieser [28] as well as Balakireva and Zamyatnin [29]). Our finding corresponded to the results presented by Comino et al. [30]. However, Comino et al. [30] found also strong cross-reactive bands at around 37 kDa, which were not detected in our study. The reason for the observed differences may lie in the fact that different oat genotypes were used in the experiments but also in different avenin extraction protocols. Densitometric analysis also revealed significant differences in the cross-reactivity of both groups in accordance with ELISA results (Figure 5C).

Thus, the genotype-specific presence or absence of avenin peptides in these reactive regions can significantly modify (in addition to the factors already mentioned) the cross-reactivity (interference) of oat cultivars with the G12 antibody. This fact is also indicated by the frequency of the occurrence of the pair of bands in 30 kDa. With the exception of the Leo cultivar, this pair of bands was detected in the whole group of oat cultivars containing relatively higher gluten-like peptide contents (≥11 mg·kg^−1^). In contrast, the frequency of the occurrence of an identical band pair was only 39% in oat cultivars with relatively low reactivity to G12 (Figure 4A,B). By appropriate selection of oat cultivars, their reactive background with the G12 antibody can thus be partially influenced.

### 3.4. Interference between Highly Concentrated Avenins of Low-Reactive Cultivars and the G12 Antibody

**Figure 6:**
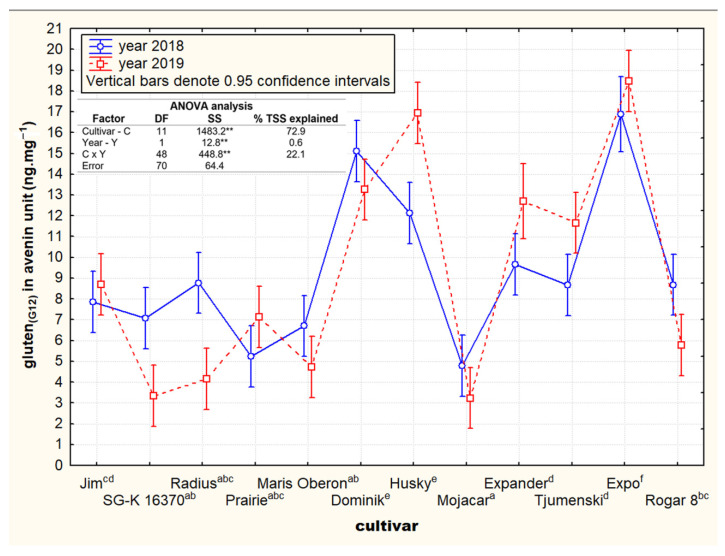
Oat cultivar differences of cross-reactive avenin peptides (gluten_(G12)_) calculated on purified avenin units in defined set of oat cultivars with the low inter-year relative standard deviation (≤30%) and the lowest average gluten_(G12)_ content (≤5 mg·kg^−1^) in dry matter of oat grain. ** significant at *p* ≤ 0.01. Note 1: Values were obtained on basis of 50-times-concentrated sample isolation compared to the standard kit procedure. Note 2: Cultivars marked with different letter apostrophes are statistically significant at *p* ≤ 0.05.

The question of a random or cultivar-specific cross-reactivity of the G12 antibody to avenin peptides was further studied in the selected 12 low-reactive oat cultivars (G12 ≤ 5 mg·kg^−1^). The 50-fold-concentrated pure avenin (see Section 2.2.3) of each oat cultivar was used as a primary sample for the immunochemical reaction with the G12 antibody using the AgraQuant ELISA kit. A two-year comparison of the detected contents of cross-reactive avenins (gluten_G12_) per pure avenin unit measured by RP-HPLC is declared in Figure 6. Similarly, Giménez et al. [31] were able to distinguish oat cultivars revealing differential quantitative amounts of G12-cross-reactive avenins based on differential RP-HPLC patterns.

The obtained results related to the contents of cross-reactive avenin peptides expressed per unit of pure avenins led to an identification of significant varietal differences but also of relatively high inter-year stability. The calculation of the Total Sum Square (TSS) based on ANOVA statistics showed that the effect of the cultivar was the main source of variation (72.9%) compared to the significant annual effect (only 0.6%) and the interaction effect of both factors (22.1%; Figure 6).

The lowest two-year average concentrations of cross reactive avenin peptides per pure avenin unit were detected in the six oat cultivars of Mojacar (4.0 ng·mg^−1^), SG-K 163702 (5.2 ng·mg^−1^), Maris Oberon (5.7 ng·mg^−1^), Praire (6.2 ng·mg^−1^), and Radius (6.5 ng·mg^−1^). The highest two-year average values showed the cultivars Dominik (14.2 ng·mg^−1^), Expo (17.8 ng·mg^−1^), and Husky (14.5 ng·mg^−1^). It is also very interesting to compare the electrophoretic variability of the bands at around 30 kDa between these two significantly different groups of cultivars. In all five cultivars with the lowest contents of cross-reactive avenins per unit of pure avenin protein, only one band occurred in this area. In contrast, the three cultivars with the highest contents of cross-reactive avenins showed two bands in the region of 30 kDa (Figure 4A).

Considering roughly 1% avenin content in the total dry grain weight and the level of reactive gluten-like epitopes as 2–4 mg kg^−1^ in the grain dry weight within the studied group of cultivars (Figure 3), the resulting levels of gluten-reactive peptides should range between 200 and 400 ng·mg^−1^ per unit of pure avenin proteins. Nevertheless, our results were not unrealistic and corresponded with the findings of Comino Montilla et al. [9] who also found a large variability in the level of cross-reactive epitopes per avenin unit (<5.4–1340 ng·mg^−1^).

There are probably several reasons for this difference. The first factor is certainly the lower recovery of pure avenins compared to the company’s standard isolation protocol. Thus, some potentially interfering peptides were not included in pure avenins. This can lead to greater inaccuracies in the assay, including a significant overestimation of cross-reactive avenin epitope contents. Additionally, the matrix of the tested sample can significantly affect the levels of immunoreactive gluten peptides as mentioned by Panda et al. [32]. Thus, even in the case of a group of cultivars with a low content of cross-reactive avenins, these results confirm significant varietal differences characterized by the presence of one resp., namely a pair of avenin bands in the 30 kDa region. In our opinion, even these follow-up results confirm that the cross-reactivity of the G12 antibody with avenins of individual oat cultivars is not completely random. In this regard, the promising work of Sajic et al. [33] has been published. The authors have developed an ELISA test which is specific for the detection of gluten from wheat, barley, and rye with no cross-reaction with the eight tested oat cultivars. One of the most accurate approaches to reactive gluten quantification should be provided by mass spectrometry-based techniques [34]. The current limitations of this approach are related to the high polymorphism of cereal proteins and the incomplete database of immunologically reactive gluten epitopes.

## 4. Conclusions

The two-year study on a set of 132 oat cultivars of different geographical origins aimed to identify oat cultivars with very low contents of gluten-like G12 antibody-reactive avenin proteins. A combined approach employing complementary techniques of G12-based ELISA, RP-HPLC, 1D SDS-PAGE, and G12-based immunoblotting led to the quantitative and qualitative determination of G12 cross-reactive avenin proteins. ELISA led to the identification of oat cultivars revealing a very low content of gluten-like avenins under the detection limit of 4 mg kg^−1^ in both years. One-dimensional SDS-PAGE revealed an association of a low content of gluten-like avenins with a specific protein band pattern in the 30 kDa region with cultivars with low gluten-reactive proteins having only one peptide band in this region, while cultivars with relatively higher contents of gluten-reactive peptides had two protein bands at this region. Due to the declared cultivar dependence and the achieved values of the content of cross-reactive avenins even over 16 mg·kg^−1^, producers of gluten-free oat products should pay attention to the selection of suitable oat cultivars. This is especially true for the possibility of producing oat-based protein concentrates, where the declared limit for celiacs could easily be falsely exceeded.

## Figures and Tables

**Table 1:** Classification of the tested set of oat cultivars according to their taxonomy and their state of origin abbreviated as a three-digit code.

Classification	Category	Number of Materials
Taxon	*A. sativa*	100
	*A. nuda*	21
	*A. sativa var. nigra*	11
State of origin	FIN	6
	CZE	46
	CAN	8
	RUS	5
	GER	17
	FRA	10
	GBR	8
	USA	7
	BEL	2
	POL	2
	AUT	6
	HUN	1
	EST	2
	IRL	2
	NOR	1
	SRB	1
	NLD	2
	ARG	2
	ITA	1
	SWE	2
	ROU	1

**Table 2:** Basic data on detected cross-reactive avenins (gluten_(G12)_) and crude protein content in the tested set of 132 oat cultivars (2018–2019).

Parameter		Mean	Min.	Max.	St. Dev.	RSD (%)
Gluten_(G12)_ (mg·kg^−1^)		7.2 *	≤4.0	17.5	3.3	46.1
Crude protein (%)		18.7	15.4	22.3	1.3	6.8
Correlation coef. Gluten_(G12)_ vs. crude protein	−0.13					

* The detected mean value 309.4 mg·kg^−1^ of Sirene cultivar from 2018 was not included.

## Data Availability

Data is contained within the article (or Supplementary Materials).

## Supplementary Materials

The following supporting information can be downloaded at: https://www.mdpi.com/article/10.3390/foods11040567/s1, Figure S1: The basic weather characteristics including the sum of precipitation and average temperature throughout 2018 and 2019 growing seasons. Table S1: A complete list of oat cultivars used in the study including their taxonomy and state of origin. Table S2: Two-year averages of oat cultivars (2018–2019) in the content of cross-reactive avenins based on standard and extrapolated calculations for the detection range below LOQ (2–4 mg·kg^−1^).
