# Supplementary figures and images for: Specific Avenin Cross-Reactivity with G12 Antibody in a Wide Range of Current Oat Cultivars
Source: Foods. 2022 Feb 16;11(4):567. doi: 10.3390/foods11040567 (PMC8871486; doi:10.3390/foods11040567)

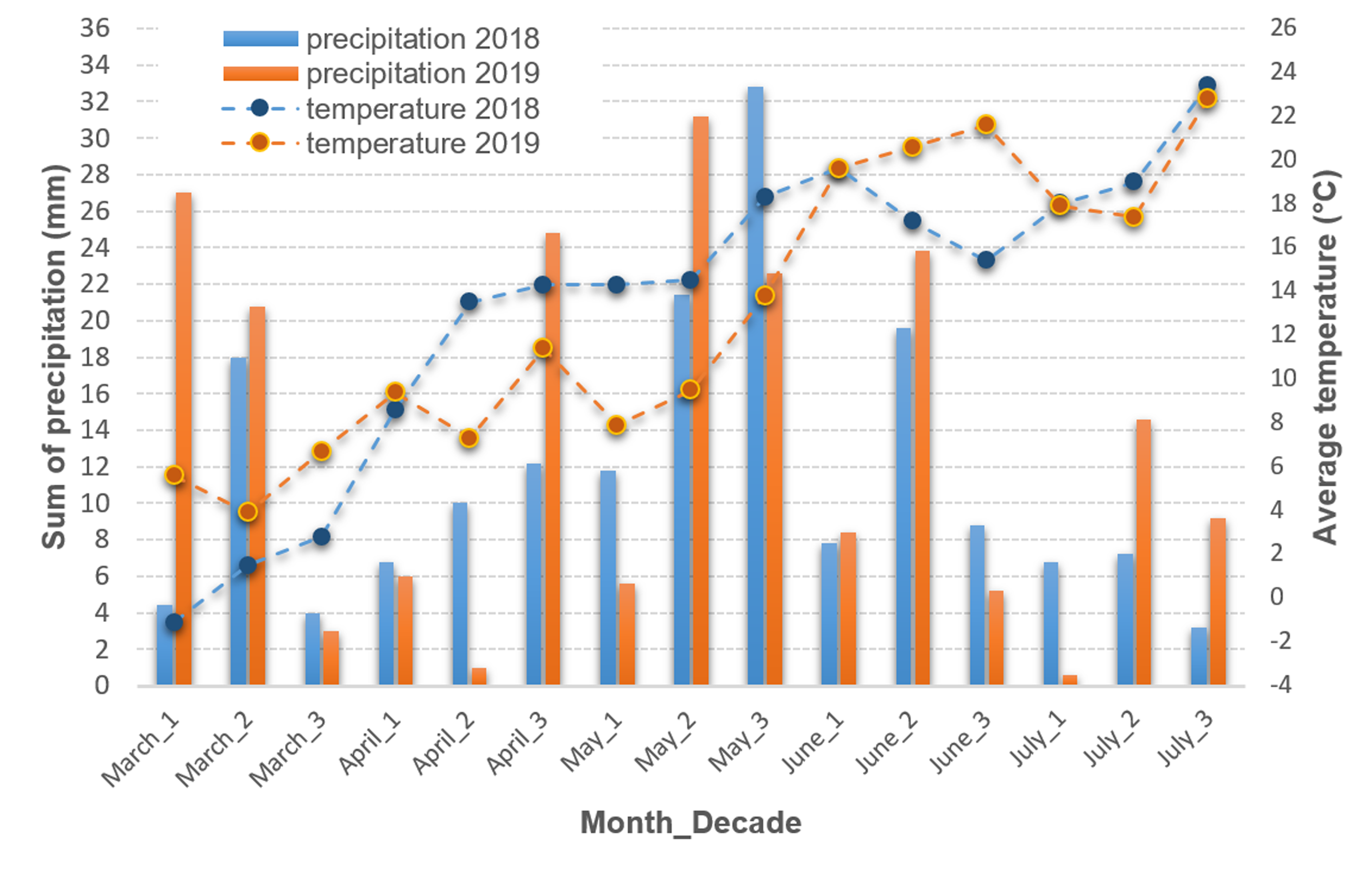

Supplement: Supplementary file 1 [file foods-11-00567-s001.zip › Suppl Figure S1.tif]
